# Phenotypic differences among and within extant populations of *Chrysanthemum arcticum* L. and *C. a.* subsp. *arcticum*

**DOI:** 10.1186/s12870-022-03902-4

**Published:** 2022-11-05

**Authors:** Yunjia Liu, Neil O. Anderson

**Affiliations:** 1 Department of Horticultural Science, University of Minnesota, St. Paul, MN 55018 USA; 2 Department of Plant Biology, The Huck Institutes of the Life Sciences, Pennsylvania State University, 101 Life Sciences Bldg., University Park, PA 16802 USA

**Keywords:** *Chrysanthemum*, Plant morphology, Population structure, Salt tolerance, Principal component analysis (PCoA), Diagnostic traits

## Abstract

**Background:**

*Chrysanthemum arcticum*, arctic daisy and its two subspecies (*Chrysanthemum arcticum* subsp. *arcticum*, *Chrysanthemum arcticum* subsp. *polaré*) are the only chrysanthemum species native to North America. A study on species’ variation in morphological and diagnostic traits is important to link morphological traits with previously described single nucleotide polymorphism (SNP) markers, particularly when the genomes are sequenced. The purpose of this study was to establish phenotypic differences and soil conditions among wild *C. arcticum* and *C. a.* subsp. *arcticum* populations, when grown in a uniform environment for two years, for potential linkages with our SNP library. Sixteen quantitative morphological traits and five qualitative morphological traits were investigated for 255 individuals from nine *C. arcticum* populations and 326 individuals from 21 *C. a.* subsp. *arcticum* populations.

**Results:**

In long-day controlled environment, *C. arcticum* flowering rate was 0% in Year 1, increased to 2.7% in Year 2, while *C. a.* subsp. *arcticum* flowering rate was 98.5% in Year 2. Two distinct clusters, distributed by taxonomic classification, were detected by Principal component analysis (PCoA) for 551 individuals from *C. arcticum* and *C. a.* subsp. *arcticum.* Pearson’s correlation coefficient analysis indicated a positive and significant correlation between plant height, flower fresh and dry weights. Flower fresh weights were correlated with Δflower weight, while inflorescence length had showed a negative correlation with leaf number. Soil samples had high Na levels along with heavy metals. Thus, the species are salt-tolerant.

**Conclusion:**

A high level of salt tolerance (Na) is tolerated by these maritime species which is a unique trait in *Chrysanthemum*. A new diagnostic trait of inflorescence length was discovered to distinguish among *C. arcticum* and *C. a.* subsp. *arcticum.* Significant flowering differences occurred among the species *C. arcticum* and *C. a.* subsp. *arcticum* under same photoperiodic environment, including flowering rates and visible bud date. This study on the species’ variation in morphological and diagnostic traits is of importance to link morphological traits with single nucleotide polymorphism (SNP) markers.

## Background

*Chrysanthemum arcticum* L., Arctic daisy (= *Arctanthemum arcticum*; = *Dendranthema arcticum*) and its two subspecies (*Chrysanthemum arcticum* subsp. *arcticum*, *Chrysanthemum arcticum* subsp. *polaré* Hultén), hereafter collectively termed the “*Chrysanthemum arcticum* species complex”, are the only chrysanthemum species native to North America [1, 2] with the center of origin and diversity in the State of Alaska (USA) and are also distributed throughout much of the maritime coastlines of Canada. Both *C. arcticum* and *C. a.* subsp. *polaré* are only found in the N. American mainland (from Alaska south and eastward in Canadian provinces), whereas *C. a.* subsp. *arcticum,* occurring both on the western Alaskan coastal mainland as well as sporadically throughout the Aleutian Islands, has two remnant populations occurring in Eurasia in the Kamchatka Peninsula (Russian Federation) and Hokkaido, Japan.

Due to taxonomic name changes and the unique position of this *C. arcticum* species complex as an evolutionary remnant from the Eurasian center of origin and diversity for the *Chrysanthemum* genus [3–6], comparative studies with other members of the genus are of great interest, particularly given the salt-tolerant nature of these N. American species. Taxa within the *Chrysanthemum arcticum* species complex share many phenotypic traits, although species-specific diagnostic traits (primarily qualitative) in the dichotomous keys inherently differentiate them [1, 7]. Leaves from both the species and subspecies are tripartite with primarily regularly toothed leaf margins whereas *C. arcticum* leaves tend to have a few more five-segmented leaves and a deep sinus. *Chrysanthemum a.* subsp. *arcticum* has leaves with a finely shallow sinus [8]. The number of midveins in the ray floret petals also differs among the species and subspecies [1, 7]. Some quantitative differentiation of the taxa within the *C. arcticum* species complex also distinguish the subspecies, e.g. *C. a.* subsp. *arcticum* plants are 10–40 cm tall whereas *C. a.* subsp. *polaré* has the shortest stems of (2.5)5–20(-26) cm [7, 9–11]. Plant height for *C. arcticum* has not been reported [1, 7, 12]. In all instances, however, these quantitative measurements, which are highly affected by factors of plant growth [13, 14], were not performed with individuals growing in a uniform environment.

Plant structure, flower and leaf architecture influence *Chrysanthemum* × *grandiflorum* and *Chrysanthemum* × *hybridum* selection and breeding for important phenotypic traits, including plant height, photoperiodic response and flower color/type [15, 16]. There are studies using multivariate analysis methods for identification within the species and populations via the morphological characteristics of the plant, including qualitative and quantitative data [17, 18]. As winter-hardy herbaceous perennials, members of the *Chrysanthemum arcticum* species complex possess advantageous phenotypic traits that do not occur in the common chrysanthemum cultivars, such as salt tolerance (growing only in coastal, maritime habitats) and a ground-cover plant habit. Unique phenotypic and genotypic features within the *C. arcticum* species complex may offer new options for transforming commercial, cultivated chrysanthemums.

We have characterized the genetic variation among extant, wild populations of *C. arcticum* and *C. a.* subsp. *arcticum,* based on 7,449 Single nucleotide polymorphism (SNP) markers from DArTseqLD [19, 20]. SNP data distinctly separated these two taxa based on population genetic structure analyses accomplished in our previous paper [19], thus providing unique SNP markers for these two species. Possible linkage of these SNP markers with phenotypic (qualitative, quantitative) traits is of great interest, particularly for species-specific traits and those of commercial interest, such as salt tolerance. The objective of this study is to establish phenotypic differences among wild *C. arcticum* and *C. a.* subsp. *arcticum* individuals when grown in a uniform environment for two years for potential linkage with SNPs [19, 20] for future research. We categorized species with representative populations (individuals as identified with SNP data) based on several phenotypic traits [18, 21, 22] as well as for native soil type composition and % survival in cultivation. The null hypothesis tested for each phenotypic trait, soil type composition or % survival was H_o_: There is no difference in phenotypic variation of each trait within and among extant populations of *C. arcticum* and *C. a.* subsp. *arcticum*.

## Results

### Pedological environment condition

The soil test results revealed a considerable disparity between the recommended greenhouse soil standards and collection sites’ samples (Table 1). The concentration of nitrate-nitrogen (NO_3_-N) from both the mainland Alaska and Attu Island collection sites were significantly lower than the greenhouse standard, especially on Attu Island which had < 0.05 ppm N. The electrical conductivity (EC) or relative dissolved soluble salt levels were in the range of 0–2 mmhos/cm (millimhos per centimeter) are non-saline, which occurred for soil samples from Ninilchik, Old Valdez-1, -2, -3, and -4 collection sites, the mainland Alaska, and the soil samples from Attu island. The Kenai-1 population had 2.1–4 mmhos/cm, or very slightly saline, whereas 4.1–8 mmhos/cm (moderately saline) was found for the soil samples from Anchor Point and Kenai-2 populations on mainland Alaska.

**Table 1:**
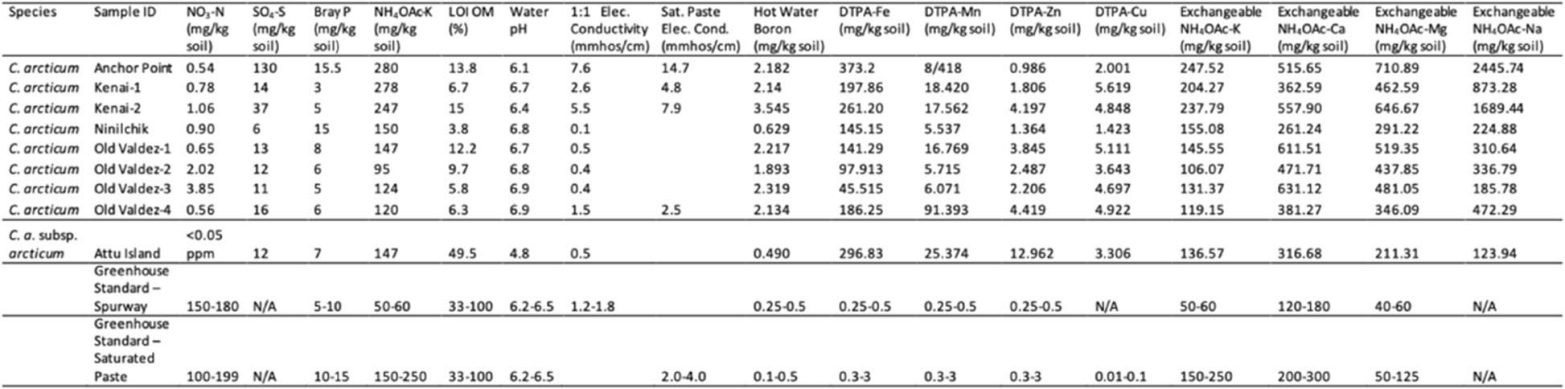
Soil test results (Spurway Greenhouse, Florist, & Nursery Crops test, Soil Testing Laboratory, University of Minnesota) [23] from select Alaskan populations of *C. arcticum* (Anchor Point-1, Kenai-1, Kenai-2, Ninilchik-1, Old Valdez-1, -2, -3, -4) and *C. a.* subsp. *arcticum* (Attu Island) with greenhouse standard for crops, including chrysanthemum

Additional soluble salt concentration and sodium (Na) level tests provided additional data on salt tolerance. The saturated paste extract EC could only be run for four populations (Table 1) due to insufficient quantities for testing. The standard reference values and relative salt tolerance of crops range from 0 to 2, 3–4, 5–7 with a maximum of 8–10 mmhos/cm. Old Valdez-4 population had the lowest of 2.5 mmhos/cm, followed by Kenai-1 at 4.8 mmhos/cm, Kenai-2 at 7.9 mmhos/cm and Anchor Point with the highest level of 14.7 mmhos/cm (Table 1). According to the soil testing laboratory, the soil sample from Old Valdez-4 would be considered slightly saline. The Kenai-1 population would be considered moderately saline whereas Kenai-2 and Anchor Point would be saline. Exchangeable NH_4_OAc-Na or sodium concentrations in all populations of both *C. arcticum* and *C. a.* subsp. *arcticum* collected were many levels of magnitude greater than the recommended greenhouse soil standard of 0–10 mg/kg (Table 1). For example, Attu Island (*C. a.* subsp. *arcticum*) had the lowest level of 123.94 mg/kg, followed by increasingly higher concentrations of Na^+^ in the *C. arcticum* populations, with the highest recorded in Anchor Point, AK at 2445.74 mg/kg (Table 1).

The water pH level of soil samples from the mainland Alaska sites ranged from 6.1 to 6.9, within the normal range for greenhouse crops, whereas Attu Island pH = 4.8 is considerably more acid (Table 1). The Bray-P test was used when the soil pH is < 7.4 (otherwise, the Olsen-P test will be used). Except for Anchor Point and Ninilchik, these soil samples collections were within the standard range as greenhouse standard (5–15 mg/kg soil; Table 1). Anchor Point and Ninilchik collection sites had higher levels of phosphorus (≥ 15 mg/kg; Table 1). The concentration of NH_4_OAc-K (mg/kg soil) of soil samples from Anchor Point (280 mg/kg), Kenai-1 (278 mg/kg), and Kenai-2 (247 mg/kg; Table 1) collection sites were greater than the range of the Greenhouse standard 75–200 mg/kg soil, while the other samples fell within this range. Extractable Zinc, Copper, Iron, and Manganese concentrations, reported as DTPA-Zn, DTPA-Cu, DTPA-Fe, and DTPA-Mn (mg/kg soil) respectively, were frequently higher than the greenhouse standards (Table 1). Meanwhile, the exchangeable Potassium, Calcium, and Magnesium concentrations were reported as Exchangeable NH_4_OAc-K, NH_4_OAc-Ca, and NH_4_OAc-Mg, respectively, also indicating high variation among the samples with exchangeable Ca and Mg having the highest range of values.

### % Survival in cultivated conditions

Since the growing requirements for species and subspecies are completely unknown, we rooted the rhizomes and grew the clones in our standard greenhouse conditions (no added Na in the soilless medium) used for cultivated chrysanthemums (as described earlier) [13, 14, 24]. The *C. arcticum* populations survived sub-optimally with all populations experiencing losses by the end of Year 2, ranging from 7.7% (Ninilchik) to 45.9% plant death (Old Valdez-1; Table 2). In contrast, all of the *C. a.* subsp. *arcticum* populations had 0% plant death (Table 2).

**Table 2:**
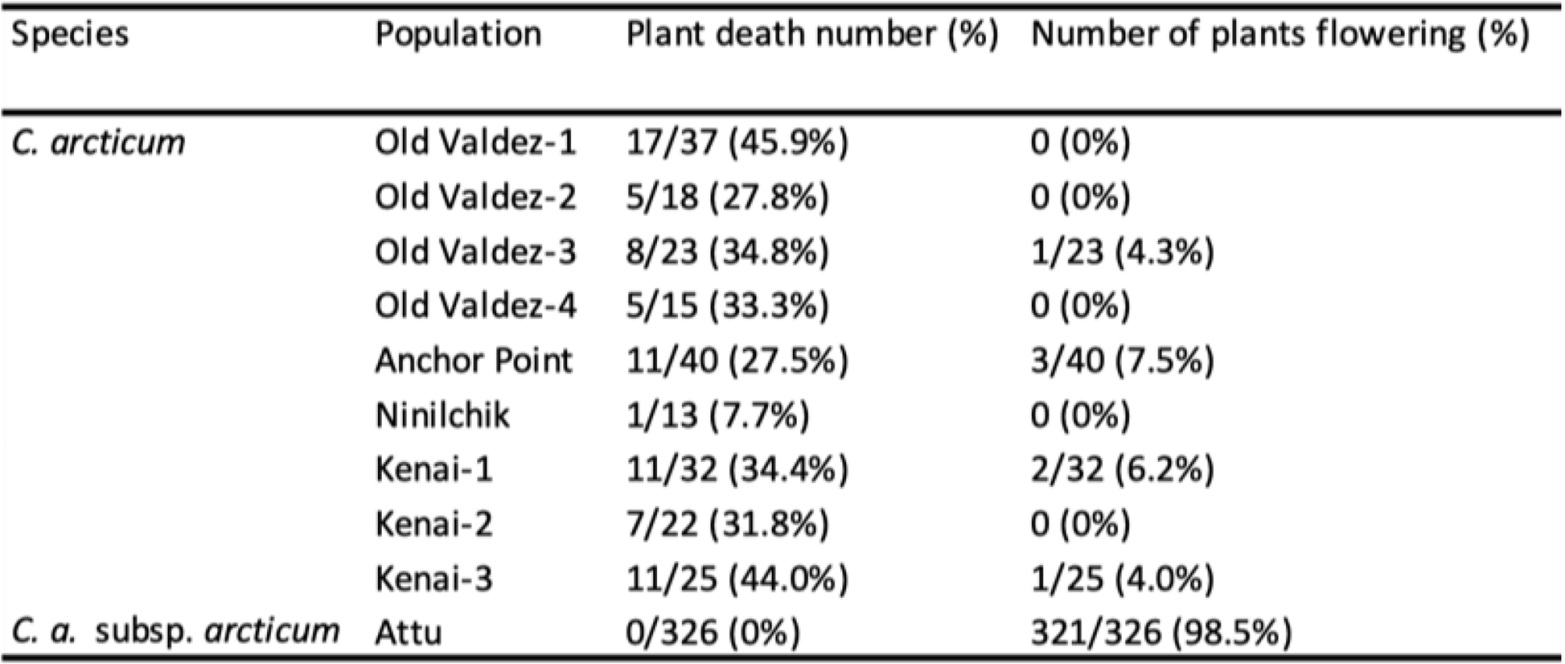
*Chrysanthemum. arcticum* (nine populations) and *C. a.* subsp. *arcticum* data for clonal ramets (rhizomes rooted from the individuals collected in the wild) grown in the greenhouse during Years 1–2 of the experimental period: plant death (number, %) and number of plants flowering (%)

### Morphological data

We observed significant differences in the majority of 21 morphological characteristics measured for *C. arcticum* species (255 individuals from nine populations) and *C. a.* subsp. *arcticum* (326 individuals from 21 populations) (Table 3). As a continuous study, all *C. arcticum* leaf morphological traits were measured in Year 2 (2019), whereas other traits were measured in Year 1 (2018; shadowed in Table 3). All *C. a.* subsp. *arcticum* morphological traits were measured in Year 2 (Table 3). Since the Attu-21 population contained < 3 individuals (n = 1) it was automatically eliminated by the SPSS program for ANOVAs. Hence, a total of 29 populations were analyzed. Except for flower fresh, dry and ΔFlower weight variables, all species, populations and species * population interactions were very highly significant (*p* ≤ *0.001)*.

**Table 3:**
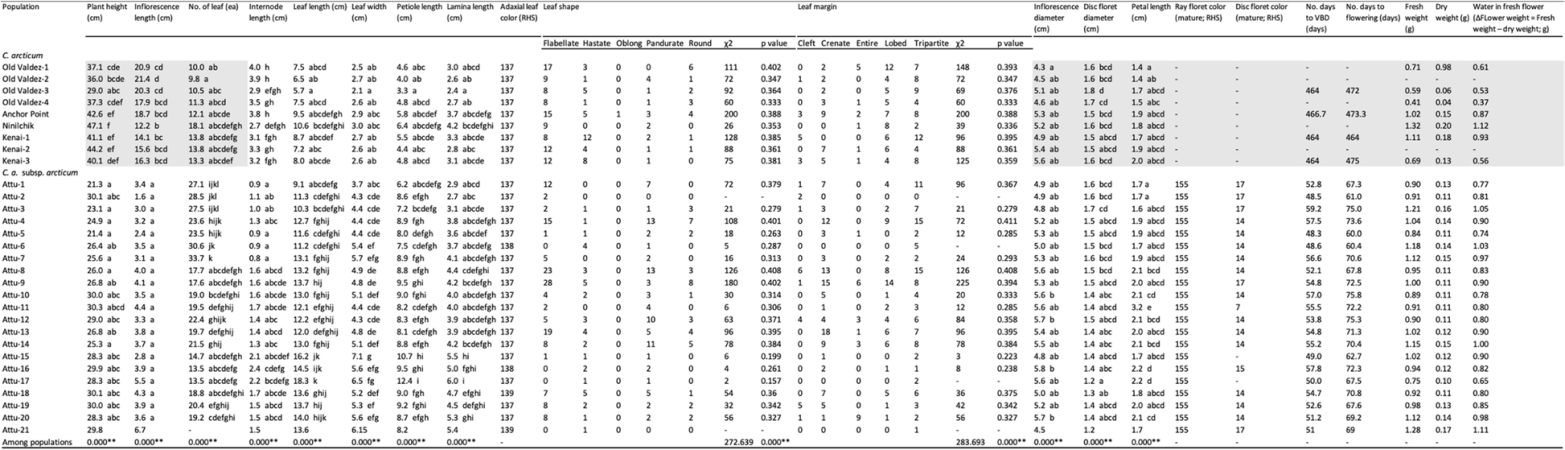
Mean values of *Chrysanthemum arcticum* (nine populations) and *C. a.* subsp. *arcticum* (21 populations) for 21 plant morphological traits (Year 1 data marked by shadow: *C. arcticum* plant height, inflorescence length, number of leaves, internode length, inflorescence diameter, disc floret diameter, flower petal length, flower fresh weight, flower dry weight and Δflower weight; Year 2 data: *C. arcticum* leaf length, leaf width, petiole length, lamina length and three qualitative traits (adaxial leaf color, leaf shape, leaf margin), number of days to visible bud date, number of days to flowering and *C. a*. subsp. *arcticum* all 21 morphological traits). Mean separations within traits (columns), are based on Tukey’s 5% HSD. Chi-square tests of two qualitative phenotypic traits (leaf shape, leaf margin) tested with equal probability of occurrence (1:1:1:1:1 χ2)

One asterisk (*) indicate a significant variation (*p* < 0.05); )

Two asterisks (**) indicate a highly significant variation (*p* < 0.01)

Mean plant height ranged from 21.3 cm (Attu-1; which overlapped with all other *C. a.* subsp. *arcticum* populations) to 47.1 cm (Ninilchik; Table 3) and there was very highly significant variation among *C. arcticum* and *C. a.* subsp. *arcticum* species (F = 11,420.89, *p* ≤ *0.001*). The *C. a.* subsp. *arcticum* populations differed significantly from *C*. *arcticum* for mean plant height, except for all Old Valdez populations (Table 3). The Ninilchik population was significantly different from all other populations of *C*. *arcticum* as well as *C. a.* subsp. *arcticum* whereas the three Kenai populations overlapped with both Ninilchik and Anchor Point. Plant height is not a diagnostic trait for these species and subspecies.

Mean inflorescence lengths, ranging from 1.6 cm (Attu-2) to 21.4 cm (Old Valdez-2; Table 3), consistently showed highly significant variation among both species and subspecies (F = 2314.247, *p* ≤ *0.001*). The shortest mean inflorescence lengths were contained in all of the *C. a.* subsp. *arcticum* populations which did not overlap with any of the *C. arcticum* (Table 3)*.* Thus, inflorescence length differs substantially between species and subspecies and is a diagnostic trait. The Ninilchik population differed significantly from only the Old Valdez-1, -2, and -3 populations but overlapped with the other *C. arcticum* populations. In contrast, there was no significant difference among *C. a.* subsp. *arcticum* populations for inflorescence length (Table 3).

The number of leaves on each primary inflorescence stem ranged from 9.8 (Old Valdez-2) in *C. arcticum* to 33.7 (Attu-7; Table 3) in *C. a.* subsp. *arcticum*. The nine *C. arcticum* populations differed significantly from Attu-1 to Attu-7 populations (*p* ≤ *0.001*). Interestingly, despite most of the *C. arcticum* subsp. *arcticum* populations having shorter plant height than *C. arcticum*, Attu-1 through Attu-6 populations had significantly greater numbers of leaves on the primary stems (Table 3). The internode length ranged from 0.8 cm (Attu-7) to 4.0 cm (Old Valdez-1; Table 3), with a highly significant difference between *C. arcticum* and *C. a.* subsp. *arcticum* species (F = 3420.38, *p* ≤ *0.001*). The Attu-1 to -7 and Attu-12 and Attu-14 populations differed significantly from all *C. arcticum* populations on the internode length morphological trait. Thus, they are distinctly different in this trait. As a result, neither leaf number nor internode lengths are diagnostic traits among species and subspecies.

Leaf lengths ranged from 5.7 cm (Old Valdez-3) to 18.3 cm (Attu-17; Table 3), while a significant difference was found between *C. arcticum* and *C. a.* subsp. *arcticum* species (F = 5199.363, *p* ≤ *0.001*). The Attu-15 to -17 populations differed significantly from the *C. arcticum* populations (Table 3). Among the *C. arcticum* populations, Old Valdez-3 and Ninilchik differed from the rest significantly (Table 3). Mean leaf widths ranged from 2.1 (Old Valdez-3) to 7.1 cm (Attu-15; Table 3) with a significant difference between *C. arcticum* and *C. a.* subsp. *arcticum* species (F = 6042.344, *p* ≤ *0.001*). *C. arcticum* populations differed significantly from all *C. a.* subsp. *arcticum* populations except for Attu-1 (Table 3). Among the *C. a.* subsp. *arcticum* populations, mean Attu-15 leaf width was significantly different from all other Attu populations, except for Attu-7, -16, -17 and -20. Mean petiole lengths, ranging from 3.3 cm (Old Valdez-3) to 12.4 cm (Attu-17; Table 3), varied significantly between the species and subspecies (F = 3977.258, *p* ≤ *0.001*). The Attu-15 and -17 populations differed significantly from all *C. arcticum* populations except for Ninilchik. Mean lamina lengths ranged from 2.4 cm (Old Valdez-3) to 6.0 cm (Attu-17; Table 3), showing highly significant variation among the species and subspecies (F = 4485.451, *p* ≤ *0.001*). Attu-15 and -17 populations differed significantly from all *C. arcticum* populations except for the Ninilchik population. Interestingly, the Attu-15 and -17 differed from the majority of *C. arcticum* populations except for Ninilchik for leaf morphological traits. Due to the outlying populations within species, e.g., Ninilchik, Attu-17, and Old Valdez-3, which caused overlap among leaf morphological traits, none of these can be identified as diagnostic.

Leaf color [25] on the adaxial surface of *C. arcticum* population was RHS 137 Green (Table 3), whereas that of *C. a.* subsp. *arcticum* populations were primarily the same color, although the Attu-6 and Attu-16 populations were RHS 138 Green whilst Attu-18 and Attu-21 populations were RHS 139 Green (Table 3). Thus, most adaxial leaf surface coloration variation occurred in *C. a.* subsp. *arcticum;* this trait is not diagnostic*.*

Most leaf shapes for the nine *C. arcticum* populations were flabellate, although a few individuals had hastate, oblong, pandurate, and round (Table 3). The Ninilchik population did not have a hastate leaf shape, although it was the second most commonly occurring leaf shape in *C. arcticum* (Table 3). The Anchor Point population was the only *C. arcticum* with oblong leaf-shaped individuals (Table 3). The *C. a.* subsp. *arcticum* Attu-2 population had a 100% flabellate leaf shape (Table 3). Unlike *C. arcticum populations, C. a.* subsp. *arcticum* did not have any oblong leaf-shaped individuals (Table 3). Hastate and pandurate leaf shapes were the most common among *C. a.* subsp. *arcticum* populations (Table 3). The 1:1:1:1:1 χ2 for leaf shape (flabellate: hastate: oblong: pandurate: round) did not differ significantly within populations (Table 3). Whereas the pooled populations 1:1:1:1:1 χ2 for leaf shape was highly significantly different (χ2 = 272.639, *p* ≤ *0.001*; Table 3) and did not fit an equal distribution. Thus, a specific leaf shape is not diagnostic of the species and subspecies.

A tripartite leaf margin was the most common type in all *C. arcticum* populations (Table 3). Except for the Attu-2 population, tripartite was the most common leaf margin in all the other *C. a.* subsp. *arcticum* populations. The 1:1:1:1:1 χ2 for leaf margin types (cleft: crenate: entire: lobed: tripartite) did not differ significantly from expected, while a highly significant difference occurred among pooled populations (χ2 = 283.693, *p* ≤ *0.001*). Since most populations, regardless of species, had one or two to five leaf margin types (cleft, crenate, entire, lobed, tripartite), this trait is not diagnostic.

*Chrysanthemum arcticum* and *C. a.* subsp. *arcticum* species were significantly different (F = 15,797.324, *p* ≤ *0.001*) for inflorescence diameter. This trait ranged from a mean of 4.3 cm (Old Valdez-1) to 5.8 cm (Attu-16; Table 3) and overlapped significantly within populations and among species. The Old Valdez-1 population was differed significantly from the Attu-10, -12, -16, and -20 populations (Table 3). Disc floret diameter ranged from 1.2 cm (Attu-17) to 1.7 cm (Old Valdez-4, Attu-3; Table 3), with highly significant variation between species and subspecies (F = 20,431.789, *p* ≤ *0.001*). The *C. a.* subsp. *arcticum* Attu-17 population was significantly different from all *C. arcticum* populations except for Kenai-1 and Kenai-2 (Table 3). Mean petal lengths were highly significantly different between *C. arcticum* and *C. a.* subsp. *arcticum* (F = 7606.809, *p* ≤ *0.001*), ranging from 1.4 cm (Old Valdez-1, Attu-1 and -2) to 3.2 cm (Attu-11; Table 3) among populations. The Attu-11 mean petal length was significantly longer from all other populations. None of these three floral traits (inflorescence diameter, disc floret diameter, and petal length) can be classified as diagnostic between the species and subspecies.

*Chrysanthemum arcticum* populations lack ray floret color and disc floret color data since there was no flower during or after plant collection in Year 1. Only seven *C. arcticum* plants flowered or reached VBD in Year 2 (Table 2), although all ray petals observed in the field were white. Ray floret colorations of all *C. arcticum* subsp. *arcticum* populations were uniformly expressed as RHS 155 white (Table 3) while the disc floret colors ranged from RHS 14 yellow to RHS 15 and RHS 17 (Table 3).

None (0%) of the *C. arcticum* individuals flowered during Year 1. However, a limited number of plants within some populations of *C. arcticum* subsequently flowered in late Year 2, long after *C. a.* subsp. *arcticum* had completed flowering. Thus, only limited flowering data is available for *C. arcticum*. Data missing from *C. arcticum* include the number of days to visible bud date (VBD) and flowering (anthesis) for most populations of *C. arcticum.* Our record showed 96.9% (218/225 individuals) did not initiate flower buds and 97.8% (220/225) did not reach anthesis during Years 1 or 2 in the greenhouse test environment (Table 2). For the seven plants that did reach VBD and/or flower at the end of Year 2, it took > 1 year; 457—486 d (65.3—69.4 wks.) to reach VBD and 462—492 d (66—70.3 wks.) to reach anthesis or flowering (Table 3), the longest period for either trait ever reported in *Chrysanthemum* [13, 14]. The seven individuals were from Anchor Point, Kenai-1, Kenai-3, and Old-Valdez-3 populations (Table 3). Thus, flowering data was collected in different years for both species and subspecies, due to the lengthy delays in *C. arcticum* flowering (Table 3)*.* In contrast with the lengthy amount of time (> 1 year) it took the seven *C. arcticum* individuals to flower, the mean number of days for *C. a.* subsp. *arcticum* individuals to reach VBD ranged from 48.33 d or 6.9 wks. (Attu-5 population) from the start of the experiment to 59.17 d or 8.45 wks. (Attu-3) under the same long-day photoperiods. The *C. a.* subsp. *arcticum* mean number of days to flowering, also termed “response group” [14], ranged from 60 d or 8.57 wks. (Attu-5) to 72.25 d or 10.3 wks. (Attu-16; Table 3). The mean duration of flower bud development from VBD to anthesis (flowering) in this species was extremely fast, taking as few as 11.67 d or 1.8 wks. (Attu-5) to 21.55 d or 3.1 wks. (Attu-12) with a pooled mean across all populations of 15.73 d or 2.2 wks..

Due to the lack of flowering in Year 2, insufficient quantities of flowers occurred in several *C. arcticum* populations. Since fresh/dry weights could not be determined with the inflorescences collected in the wild in Year 1 (due to seed ripening to obtain open-pollinated progeny), most of the flower weight data are missing (Table 3). The mean fresh weight of the flowers ranged from 0.59 g (Old Valdez-3) to 1.28 g (Attu-21; Table 3). In contrast, water loss created mean dry weights ranging from 0.06 g (Old Valdez-3) to 0.17 g (Attu-21; Table 3) / inflorescence. The ΔFlower weight (Fresh weight minus dry weight) values ranged from 0.53 g (Old Valdez-3) to 1.11 g (Attu-21; Table 3). None of these traits would be diagnostic for the species and subspecies.

Pairwise correlations among 16 quantitative variables were primarily positively and significantly correlated (Table 4). Plant height was significantly and positively correlated with inflorescence length (r = 0.610), the number of leaves (r = 0.311), disc diameter (r = 0.207), number of days to VBD (r = 0.366) and flowering (r = 0.315), fresh (r = 0.157) and dry weights (r = 0.196; Table 4). Plant height also had a highly significant negative correlation with internode length (r = -0.509), leaf length (r = -0.329), and leaf width (r = -0.466; Table 4). Inflorescence length was positively and significantly correlated with the number of days to VBD (r = 0.644) and flowering (r = 0.678) but negatively and significantly correlated with internode length (r = -0.718), number of leaves (r = -0.604), leaf length (r = -0.576), leaf width (r = -0.651), petiole length (r = -0.605), lamina length (r = -0.380), and inflorescence diameter (r = -0.199). Inflorescence length was positively correlated with flower dry weight (r = 0.034), while negatively correlated with flower fresh weight (r = -0.040) and ΔFlower weight (r = -0.051; Table 4). Overall, plant height and inflorescence length were all negatively correlated with all four quantitative leaf morphological traits (Table 4). The number of leaves was significantly and positively correlated with internode length (r = 0.210), leaf length (r = 0.286), leaf width (r = 0.326), petiole length (r = 0.321), lamina length (r = 0.142) but negatively correlated with number of days to VBD (r = -0.180) and flowering (r = -0.177; Table 4). Internode lengths were significantly and positively correlated with leaf length (r = 0.610), leaf width (r = 0.684), petiole length (r = 0.624), lamina length (r = 0.444), and flower petal length (r = 0.313) but negatively correlated with number of days to VBD (r = -0.365) and flowering (r = -0.295; Table 4). Interestingly, the internode length was highly significantly and positively correlated with inflorescence diameter (r = 0.204) whereas it was negatively correlated with disc floret length (r = -0.326).

**Table 4:**
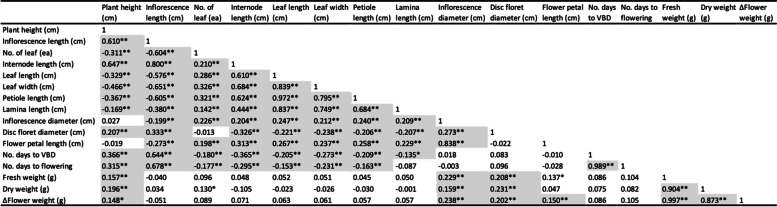
Correlations between 16 quantitative plant morphological traits for *C. arcticum* (225 individuals) and *C. arcticum* subsp. *arcticum* (326 individuals) (2018 data: *C. arcticum* plant height, inflorescence length, number of leaves, internode length, inflorescence diameter, disc floret diameter, flower petal length, flower fresh weight, flower dry weight and Δflower weight; 2019 data: *C. arcticum* leaf length, leaf width, petiole length, lamina length, number of days to visible bud date, number of days to flowering; and *C. a.* subsp. *arcticum* all 16 quantitative morphological traits)

An asterisk (*) indicates a significant correlation coefficient (*P* ≤ *0.05*)

two asterisks (**) indicate a highly significant correlation coefficient (*P* ≤ *0.01*) whereas a lack of any asterisk(s) denotes not significant

Leaf length was highly correlated with leaf width (r = 0.839), petiole length (r = 0.972), lamina length (r = 0.837), but not as correlated with flower diameter (r = 0.247), flower petal length (r = 0.267), flower fresh weight (r = 0.052), and ΔFlower weight (r = 0.063; Table 4). Leaf length was negatively correlated with disc diameter (r = -0.221), number of days to VBD (r = -0.205) and flowering (r = -0.153). Leaf width was positively correlated with petiole length (r = 0.795), lamina length (r = 0.749), inflorescence diameter (r = 0.212), and flower petal length (r = 0.237), while negatively correlated with disc diameter (r = -0.238), number of days to VBD (r = -0.273), flowering (r = -0.231). Leaf width was positively correlated with flower fresh weight (r = 0.051) and ΔFlower weight (r = 0.061), but negatively correlated with dry weight (r = -0.026). Petiole length was positively correlated with lamina length (r = 0.684), inflorescence diameter (r = 0.240), while highly significant and negatively correlated with disc diameter (r = -0.206), number of days to VBD (r = -0.209) and flowering (r = -0.163). Petiole length was positively correlated with flower fresh weight (r = 0.045), and ΔFlower weight (r = 0.057), but negatively correlated with dry weight (r = 0.030). Lamina length was highly significant and positively correlated with inflorescence diameter (r = 0.209), flower petal length (r = 0.229); was highly significant and negatively correlated with disc diameter (r = -0.207). Lamina length was positively correlated with flower fresh weight (r = 0.050), and ΔFlower weight (r = 0.057), while negatively correlated with dry weight (r = -0.001).

All four quantitative leaf morphological traits (leaf length, leaf width, petiole length and lamina length) were very significant and highly positively correlated (Table 4). In contrast, all quantitative leaf morphological traits were negatively correlated to disc floret diameter, the number of days to VBD, and flowering (except for lamina length), respectively (Table 4). Additionally, these four quantitative leaf morphological traits were positively correlated with fresh weights and ΔFlower weight while negatively correlated with dry weights.

The inflorescence diameter was positively and significantly correlated with all other floral traits (disc floret diameter, r = 0.273; petal length, r = 0.838; fresh weight, r = 0.229; dry weights r = 0.159; and ΔFlower weight, r = 0.238)) while negatively but not significantly correlated with the number of days to flowering (r = -0.003; Table 4). The disc floret diameter was significantly and positively correlated with flower fresh (r = 0.208), dry weights (r = 0.231) and ΔFlower weight (r = 0.202), whereas it was negatively correlated with flower petal length (r = -0.022). Flower petal length was significantly positively correlated with inflorescence diameter (r = 0.838) and negatively correlated with disc floret diameter (r = -0.022). The flower petal length was positively correlated with ΔFlower weight (r = 0.150) and fresh weights (r = 0.137) while negatively correlated with the number of days to VBD (r = -0.010) and flowering (r = -0.028; Table 4). The number of days to VBD was positively correlated with the number of days to flowering (r = 0.989), and positively correlated with fresh (r = 0.086) and dry weights (r = 0.075) and ΔFlower weight (r = 0.086). The number of days to flowering was positively correlated with flower fresh weight (r = 0.104), dry weight (r = 0.082) and ΔFlower weight (r = 0.105). Fresh and dry weights and ΔFlower weight traits were significantly and positively correlated with each other, as would be expected.

### Principal components analyses (PCoA)

**Fig. 1:**
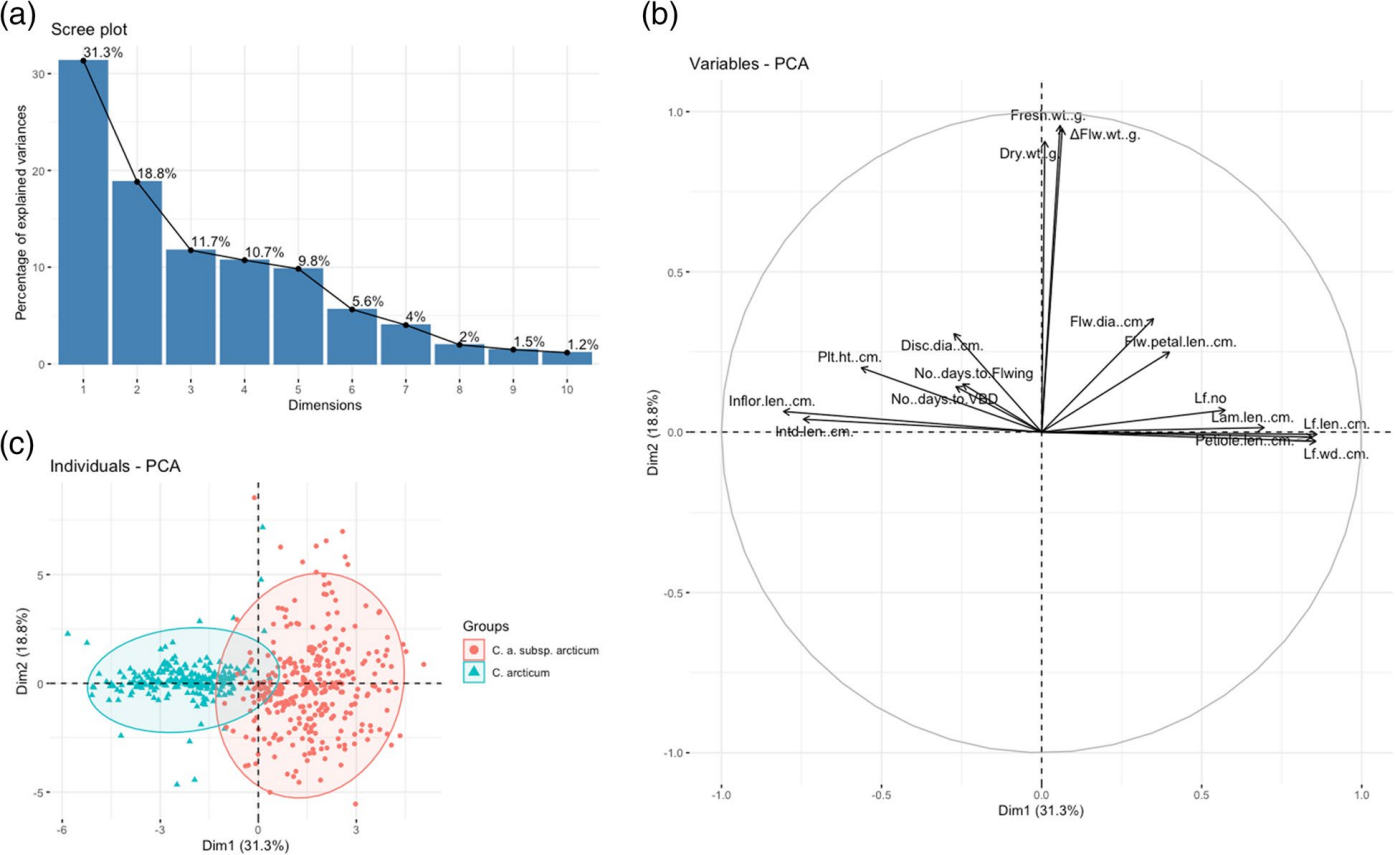
Principal components analysis (PCoA) for *Chrysanthemum arcticum* and *C. a.* subsp. *arcticum* for 16 quantitative morphological traits. **a** Scree plot of principal component analysis of *C. arcticum* populations between eigen value and principal components; **b** variables plot revealed by two principal components analysis; **c** Individual scatter plot grouping by species with two principal components analyses

For Group one (both species and subspecies analyzed together), the first two principal components (PC1 and PC2) accounted for 50.1% of the variation (Fig. 1a). PC1 accounted for 31.3% of the total variation and was positively associated with the number of leaves, leaf length, petiole length, lamina length and leaf width, ray floret diameter, flower petal length, flower fresh weight, dry weight, and ΔFlower weight. PC1 was negatively associated with disc floret diameter, plant height, inflorescence length, internode length and the number of days to VBD and flowering (Fig. 1b). PC2 accounted for 18.8% of the total variation. PC2 was positively associated with most morphological variables, but negatively associated with leaf and petiole length and leaf width (Fig. 1b). The variable biplot revealed that fresh and dry weights and ΔFlower weight variables were closely clustered; leaf number, leaf length, petiole length, leaf width and lamina length were clustered together. All individuals were categorized into two groups into *C. arcticum* and *C. a.* subsp. *arcticum* species (Fig. 1c). In the scatter plot, two clusters showed overlapping distribution yet separated distinctly along the PC1 for 30.1% of the total variance. Compared with *C. arcticum*, the *C. a.* subsp. *arcticum* group was dispersed widely along the PC2 for 18.8% of the total variance and presented more outliers from the ellipses.

**Fig. 2:**
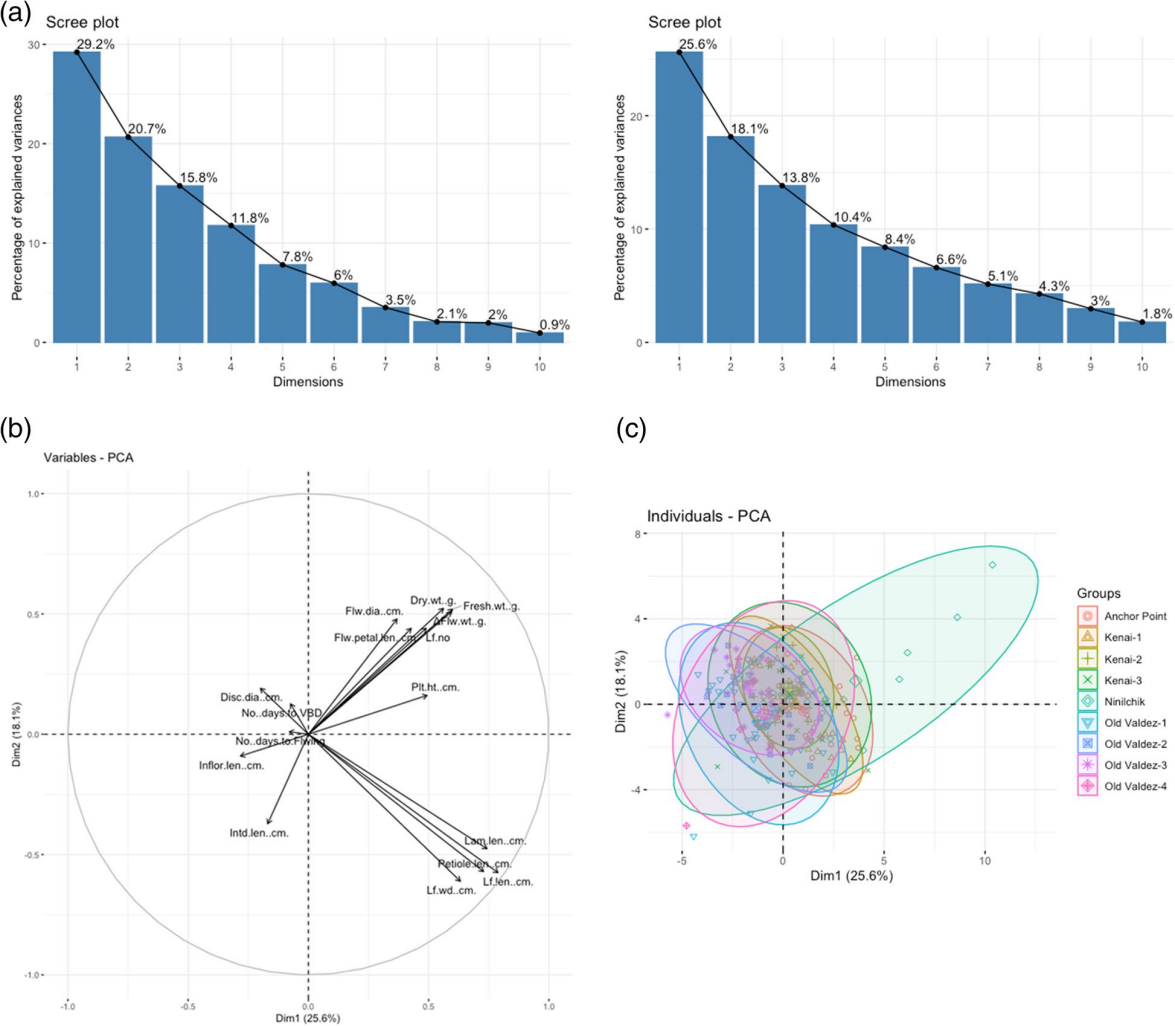
Principal components analyses (PCoA) for *Chrysanthemum arcticum* 16 quantitative morphological traits. **a** Variables plot revealed by two principal components analysis. **b** Scree plot of principal component analysis of *C. arcticum* populations between eigen value and principal components. **c** Individual scatter plot grouping by populations revealed by two principal components analysis

For Group two, PCoA of 16 morphological variables of the nine populations of *C. arcticum*, 10 principal components were determined with 100% cumulative contribution (Fig. 2a). The first two principal components for Group two analysis, PC1 and PC2 accounted for 43.7% of total variation (Fig. 2b). PC1 accounted for 25.6% of the total variance. It was positively associated with leaf length, petiole length and lamina length, leaf width, plant height, flower diameter, flower petal length and all three flower weight characteristics. However, it was negatively associated with inflorescence length, flower disc diameter, internode length, and number of days to VBD and flowering. PC2 accounted for 18.1% of the total variance. It was positively correlated with leaf width and length, petiole length and lamina length, internode length and inflorescence height, number of days to VBD and flowering. However, it was negatively correlated with inflorescence diameter, disc diameter, plant height, flower petal length, number of leaves, fresh and dry weights, and ΔFlower weight. Four quantitative morphological leaf variables were closely clustered together positively associated with PC1 and negatively associated with PC2. Fresh, dry weights, and ΔFlower weights were clustered closely, associated with both PC1 and PC2 positively. However, instead of the relationship between species and subspecies, *C. arcticum* revealed a highly mixed distribution among the nine populations based on a multivariate analysis of the morphological characteristics. According to the scatter plot (Fig. 2c), the Ninilchik population had a wider range of variation than other populations for *C. arcticum* species. Four individuals from the Ninilchik population were outliers from the overlapping distribution, along with three individuals from Old Valdez-1, Old Valdez-3, and Old Valdez-4.

**Fig. 3:**
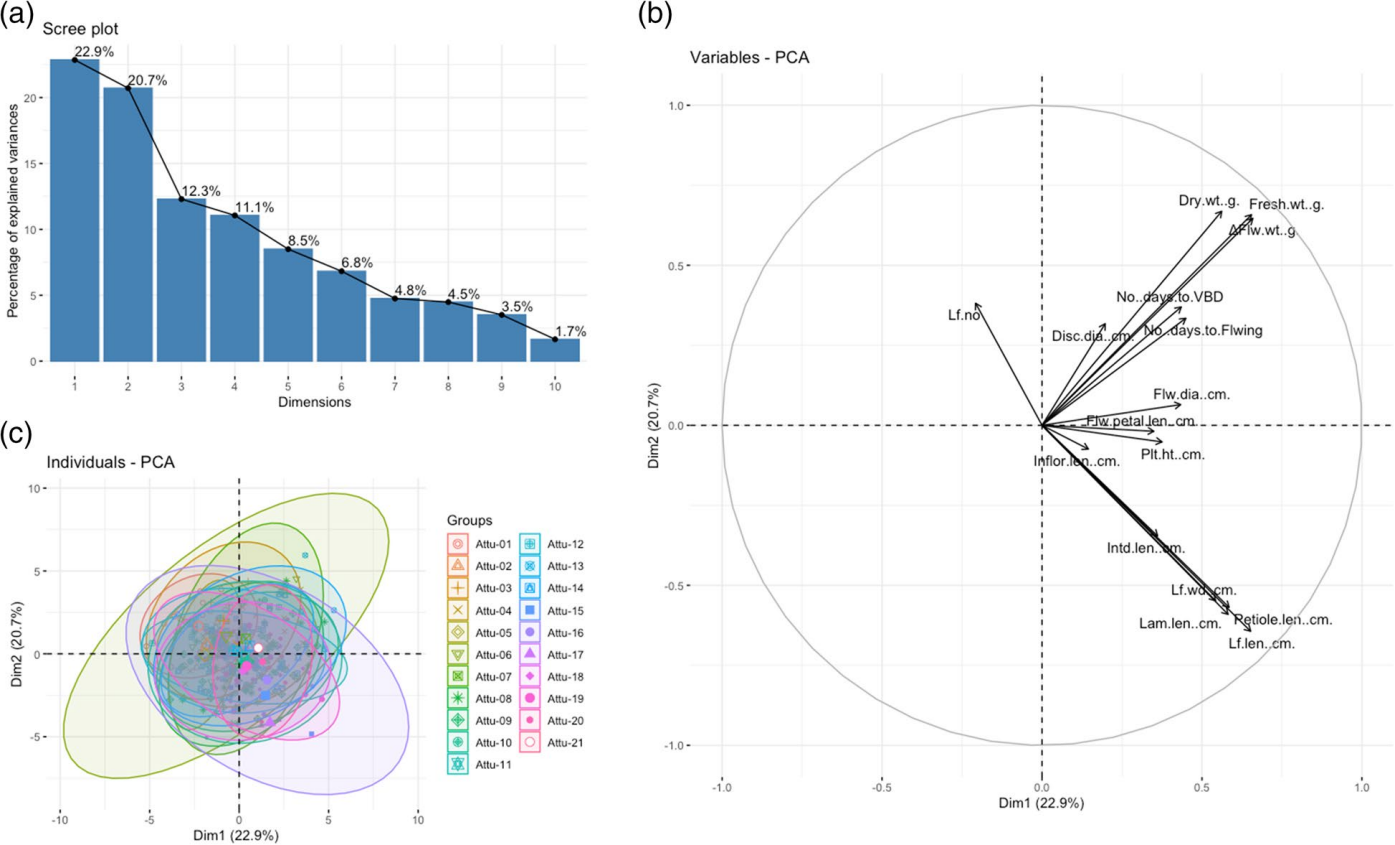
Principal components analyses (PCoA) for *Chrysanthemum arcticum* subsp. *arcticum* 16 quantitative morphological traits. **a** Variables plot revealed by two principal components analysis. **b** Scree plot of principal component analysis of *C. a.* subsp. *arcticum* populations between eigen value and principal components. **c** Individual scatter plot grouping by populations revealed by two principal components analysis

In contrast, Group three PCoA were relatively indistinguishable for the first two principal components (Fig. 3a) compared with Group one (Fig. 1a) and two (Fig. 2a) PCoAs. The first two principal components accounted for 43.6% of total variation derived from 16 quantitative morphological traits in the 21 populations of *C. a.* subsp. *arcticum* (Fig. 3a). PC1 accounted for 22.9% of the total variance and was positively associated with all variables except for the number of leaves. PC2 accounted for 20.7% of total variance. It was highly positively associated with fresh and dry weights, ΔFlower weight, number of days to VBD, flowering, the number of leaves, inflorescence and disc floret diameters, and flower petal length (Fig. 3b). It was negatively associated with leaf, petiole, lamina length and leaf width, plant height and inflorescence length (Fig. 3b). The fresh and dry weights and ΔFlower weight variables were closely clustered together, which were positively associated with both PC1 and PC2. The leaf length, leaf width, petiole and lamina lengths also clustered closely, positively associated with PC1 but negatively with PC2. Similar to the Group two analysis, the *C. arcticum* subsp. *arcticum* individual scatters plot showed a highly overlapping distribution among the 21 populations, based on the morphological characteristics. Individuals within populations tended to disperse along the PC1 instead of PC2.

## Discussion

### Examined salt-tolerant traits and potential cultivated uses

Soil test results showed lowered N levels across species and subspecies collection sites (Table 1), consistent with heavy precipitation level. The precipitation levels ranged from 72.6 mm to 168.4 mm per year in Attu Island, while 67.6 mm to 228.9 mm per year at Old Valdez, 18.8 mm to 84.6 mm per year at Kenai, 25.4 mm to 75.7 mm per year at Anchor Point, and 23.9 mm to 86.6 mm per year at Ninilchik [26]. However, this doesn’t mean that either species or subspecies are low N feeders. It has no evidence that N toxicity occurred in either species or subspecies despite being fed 125 ppm N as constant liquid feed (see Materials & Methods) used for commercial chrysanthemums [14, 27]. Thus, these *Chrysanthemum* species utilize available nitrate N (NO_3_^−^) during the growth phases.

Soil pH levels among species and subspecies may be a physical diagnostic trait since all *C. arcticum* populations grow naturally in the standard soil pH range for the genus (pH = 6.2—6.8; Table 1) [14, 27] *C. a.* subsp. *arcticum* populations grew in acidic soils at a pH = 4.8 (Table 1). The collection sites on Attu Island had high concentrations of sphagnum moss on the soil surface, regardless of whether they were on cliff faces by the ocean or adjacent shoreline areas. This sphagnum moss favors lower pH levels. Although precipitation falls as rain or snow are similar constantly happened on Attu Island and Alaska mainland, the other prerequisites of collection sites may cause different predominance species and then lead to the pH variation. Despite the acid-tolerant trait for *C. a.* subsp. *arcticum,* no evidence of nutrient deficiencies occurred in any individuals during Years 1–2 of the experimental period [27] when the soil pH was maintained within the pH = 6.2–6.8 range. Thus, the pH tolerance of *C. a.* subsp. *arcticum* has a wider range than that found in the Attu Island soil.

While most of the EC levels were within recommended low soluble salts (SS) ranges, both Anchor Point and Kenai-2 locations were in the saline range (Table 1). However, since these tests only measure SS, rather than Na levels in the soils, the NH_4_OAc-Na amounts showed all soil sample sites to be excessively high in Na, in contrast with the greenhouse recommended standard (Table 1) [27]. Even the lowest levels at Attu Island (123.94 mg/kg Na) were high but surpassed by the increasingly higher amounts in the maritime sites on the Alaskan mainland where as much as 2445.74 mg/kg was found at Anchor Point, AK (Table 1). These incredibly high levels of Na in the soils adjacent to the ocean indicate a high level of salt tolerance in both species and subspecies which is unusual for any other chrysanthemum species [13, 14]. This trait would be of significant interest to chrysanthemum breeding programs throughout the world, providing options for growing chrysanthemums in locations with saline water and saline soils. The improved salt tolerant cultivars can expand commercial chrysanthemum growing distribution and time, such as along roadways in northern latitudes where salt is used for ice melt in the winter months, as well as saline conditions in the desert southwest. Future studies will be devoted to analyzing the levels of salt tolerance in these species and subspecies and understanding the mechanism(s) involved.

While the levels of P and K were in the recommended range among the soil samples (Table 1), Anchor Point and Kenai-1 and -2 collection sites were high in K. Other nutrients, such as Zn, Cu, Fe, Mn, Ca, and Mg, were frequently higher than the norms (Table 1) [27] It was observed, particularly in the Attu Island sites, that significant WWII military waste may have seeped into the soils given the predominance of oil barrel dumps throughout the island where the 21 populations of *C. a.* subsp. *arcticum* were collected. Such tolerance to these micronutrients and heavy metals may indicate the potential use of either species or subspecies in soil mitigation. Future studies will examine the levels of heavy metals in the leaves of Arctic daisies to determine whether they are sequestered therein.

It was noteworthy that high mortality rates of 7.7% to 45.9% among the *C. arcticum* populations and 0% mortality among all *C. a.* subsp. *arcticum* populations (Table 2). Notably, the pH range of greenhouse irrigation water falls into the pH range of native soil for all collection sites, which indicates pH might not be related to the mortality rates. We observed that the dead individuals had root rot from unknown pathogens despite having routine applications of fungicide rotations applied. The specific reasons for this high mortality are unknown and deserve attention in future research.

### *C. arcticum* and *C. a. *subsp. *arcticum* taxonomic identification

In this study, we integrated conventional multivariate analysis techniques for 16 quantitative and five qualitative morphological characteristics for 525 individuals (Group one) to determine phenotypic differences between *C. arcticum* and *C. a.* subsp. *arcticum* and among populations within species and subspecies. The phenotypic data we collected in Years 1 (plant height, inflorescence length, number of leaves on the primary stem, internode length, inflorescence diameter, disc floret diameter, petal length, flower fresh weight, flower dry weight, water in fresh flower) and 2 (leaf morphology, leaf length, lamina length, petiole length, leaf width, leaf margin, shape and color) were consistent with the historical record for *C. arcticum* and *C. a.* subsp. *arcticum* and were applied to identify the variation between the species and subspecies [1, 8, 28, 29]*.* According to Nishikawa and Kobayashi [8], *C. a.* subsp. *arcticum* leaves tend to have a finely shallow sinus compared with *C. arcticum* species’ deep and regularly toothed leaf margins. Our collection (Table 3), as expected, matched the historic taxonomic records. *C. a.* subsp. *arcticum* populations tend to have more crenate leaf margins, while *C. arcticum* had more tripartite and lobed leaf margins (Table 3). The significant variation pooled among populations revealed by the Chi-square test for leaf margins indicated the diagnostic difference between *C. arcticum* and *C. a.* subsp. *arcticum* species (Table 4). Consistently, it was found in the Group one PCoA that *C. arcticum* and *C. a.* subsp. *arcticum* were primarily separated as taxa (Fig. 1c). This distinguishable classification agrees with the genetic variation of SNP markers between *C. arcticum* and *C. a.* subsp. *arcticum* found previously [19, 20]. The common morphological traits that *C. arcticum* and *C. a.* subsp. *arcticum* shared could be related to the overlapping distribution on the individual scatter plot for all individuals. However, this relatedness may be decreased due to the lack of gene flow between species, caused by the restrictive geographical separation [19, 20].

Among the collective morphological traits, it was notable that plant height of the Attu Island *C. a.* subsp. *arcticum* populations were all significantly shorter than most *C. arcticum* populations (Table 3), with the exception of all Old Valdez populations. The reason is unknown but it could be an evolutionary adaptive change. All populations are reproductively isolated due to highly mountainous terrain, although inbreeding depression could also be possible. Other morphological traits recorded did not prove to be distinguishing diagnostic characteristics between the species and subspecies (Table 3). Frequently, related leaf or floral traits were highly and significantly correlated with each other, as would be expected (Table 4). The number of leaves is similar in range to those reported for cultivated *C.* × *grandiflorum* and *C.* × *hybridum* [24]. Inflorescence length is a diagnostic trait, based on the significant differences among the species and subspecies with all individuals of *C. a.* subsp. *arcticum* having significantly shorter inflorescences than all *C. arcticum* (Table 3).

As noted earlier, the morphological variation among populations may not be distinguished significantly based on principal components analysis, although taxa differed significantly for specific traits. This may be associated with a close relatedness for individuals within and among populations. Meanwhile, the morphological variables we selected could fall into the common traits shared for species and subspecies or indistinguishable enough as diagnostic traits. However, the univariate analysis of variance provided a significant variation among populations consistent with our previous genetic variation studies. For example, for the *C. arcticum* extant populations, Old Valdez and Kenai populations tended to be significantly different based on most variables (Fig. 2): inflorescence length, number of leaves, internode length, inflorescence diameter, flower petal length. In contrast, Anchor Point and Ninilchik populations showed an intermediate tendency between Old Valdez and Kenai populations. The ANOVA among populations of *C. arcticum* showed good consistency with our previous genetic structure analyses, suggesting that the variation between Old Valdez populations cluster and Kenai populations cluster contributed to the most significant variation among populations within the species. The variance among Anchor Point and Ninilchik populations contributed to the total variance secondarily (*cf.* PCoA analyses [19]).

### Phenotypic variation matches previous genetic studies

The phenotypic variation noted in this study agrees with the genetic relatedness revealed in the UPGMA phylogenetic tree (based on using Jaccard genetic distance) [19, 20]. A detailed genetic distance within each collection sites, such as for Old Valdez -1 and -2 populations in the same genetic ward whilst Old Valdez -3 and -4 populations were successively distant related [19, 20]. The phenotypic variation obtained from this study in agreement with the genetic relatedness; Old Valdez populations, especially for the Old Valdez -1 and -2 groupings, are very significantly different from other populations for the majority of morphological traits (Table 4). Additionally, the variance among populations based on morphological characteristics reconfirmed that geographical isolation might be the major reason leading to the genetic and phenotypic variation among populations within *C. arcticum*. Likewise, it is possible that the environmental history of the collected rhizomes from the wild may have impacted the phenotypes observed in this uniform environment, although future studies would be required to test this non-genetic effect.

In the ANOVAs, *C. a.* subsp. *arcticum* populations differed significantly from *C. arcticum* populations for most morphological traits, although the range in variation within and among populations and species created an overlap of many morphological traits (Table 3). This might be expected since similarly indistinct clustering with the SNP cluster analysis for *C. a.* subsp. *arcticum* occurred*,* which verified the possibility of more frequency gene flow among Attu Island collection sites [19, 20]. The Attu populations’ geographical distributions were not as distant as the *C. arcticum* populations were from each other. However, the UPGMA phylogenetic tree from SNPs revealed a detailed genetic distance among populations and closer genetic groupings [19, 20], consistent with some phenotypic relationships among populations (Table 4; Fig. 3). For example, the Attu-8 and Attu-9 populations differed from Attu-1 to -7 populations significantly for internode length, leaf length, leaf width, which agrees with the Attu-8 and Attu-9 SNP populations groupings in the phylogenetic tree [19, 20]. Interestingly, the Attu-15, -16 and -17 populations differed significantly from other populations for the majority of morphological traits whereas the genetic SNP analysis did not present significant variation between these groupings and other populations [19, 20]. Testing these populations in additional environments would provide useful data on genotype x environment interactions or the stability of trait expression.

Correlations showed that plant height was positively related to all the flower morphological variables except flower petal length (including inflorescence length, inflorescence diameter, disc floret diameter, flower weight), which confirmed a robust vegetative growing would benefit the reproductive growth (Table 4). As expected, leaf morphology showed a significantly positive correlation among leaf variables, such as leaf, petiole, lamina lengths and leaf width for both *C. arcticum* and *C. a.* subsp. *arcticum* (Table 4)*.* Floral morphological traits were also interrelated with significantly positive correlations among variables: inflorescence diameter, disc diameter, flower petal length, and a series of flower weight characteristics (Table 4). Other pairs of variables were inevitably correlated, such as inflorescence diameter and fresh weight (Table 4). As noted before, more morphological traits will be considered in future research, such as pollen and seed morphological characteristics, presence of chemical compounds such as pyrethrin, ploidy and/or reproductive barrier(s) [6, 18, 30]. A thorough cytological study would be useful with this expansive germplasm collection within the *C. arcticum* species complex, since reported ploidy levels differ and differing levels may be diagnostic traits for the species and subspecies [31].

### *Chrysanthemum arcticum, C. a.* subsp. *arcticum* reproductive and conservation resources

Previous studies on chrysanthemum species and cultivar variation based on morphological characteristics, tended to focus on specific morphological traits with ornamental market value, such as inflorescence morphology and chemical composition [30, 32–34] or descriptive traits for U.S. plant patents or plant breeder’s rights [22, 24]. Our extensive morphological data sets, especially on qualitative and quantitative traits that best discriminate between species and populations of *C*. *arcticum, C. a.* subsp. *arcticum* and *C. a.* subsp. *polaré* is a valuable resource for future research. This morphological dataset will be enhanced with additional traits to facilitate the identification of phenotypes among species and populations and provide opportunities for marker assisted selection.

Throughout the distributional range of *C. arcticum* and *C. a.* subsp. *arcticum* flower during the summer months (long-day photoperiods), so the species are considered long-day plants. As expected, our record showed all *C. arcticum* plants were at peak flowering in late July 2018 during the collection trip, which means they would have initiated and developed flower buds during long-day photoperiods. Further, our data with greenhouse forcing confirm this particularly well with *C. a.* subsp. *arcticum* (Table 4), ~ 100% (98.5%) of which flowered in both years under long-day conditions (16 h photoperiod; Table 3). The significant lack of flowering within all populations of *C. arcticum* over a two-year period in the present study (Years 1–2; Table 3) is curious. While a few individuals reached VBD (3.1%; Table 2) and flowered (2.2%) successfully, it was after ~ 1.5 years under inductive long-day photoperiods. Clearly, another factor(s) of plant growth is required for *C. arcticum* to reach VBD and flower successfully, as occurs in the wild. We postulate that Na levels in the soil or salt spray along the oceanic coasts may be a potential primary factor in the flowering process for this species. Future research will be devoted to this question to understand the factor(s), particularly Na, and physiological mechanisms of this unusual phenomenon within *C. arcticum.* The long-day flowering in the *Chrysanthemum arcticum* complex germplasm is the opposite of what is found in cultivated *C.* × *grandiflorum* and *C.* × *hybridum* [14, 24] which are short-day plants (8 h photoperiod). However, *C. arcticum* and *C. arcticum* subsp. *arcticum,* as long-day plants, would be similar to some other chrysanthemum species, such as pyrethrin, *C. cinerariifolium* [35].

By wk. 29 (Year 1), all of the *C. arcticum* plants in the wild were at peak flowering (Anderson N. O., unpublished data) and, based on field observations as well as greenhouse trials in the current experiment, neither species re-flowers in the same season. Thus, the observed flowering period was shorter than previously reported for either species or subspecies, e.g., flowering was noted in historical specimens to occur from May 21 (wk. 21) to September 25 (wk. 39) during the growing season across the geographical distributional range [19, 20]. This shortened flowering period was assumed to be related to environmental factors, possibly global warming temperatures, that may have caused widespread extinction of the species since they were reported as “common” in the historical records (as far back as 1865; N. Anderson, unpublished data) as well as taxonomic reports [36]. This trend agrees with the previous warming simulation studies [37], which showed major northward shifts and significant reductions of the tundra biomes in the Arctic, becoming restricted to coastal and mountainous areas [38].

From the perspective of conservation, future research will launch analogous analyses on our extensive collection of herbarium specimens on morphological traits for *C. arcticum*, *C. a.* subsp. *arcticum* and *C. a.* subsp. *polaré* and other related species, which will contribute to determine the extent and magnitude of a potential genetic bottleneck in the species occurring over time. Likewise, a morphological and genetic (SNP) study of *C. a.* subsp. *polaré* populations in Alaska and Canada will be possible as soon as extant populations are collected to confirm whether this subspecies is similar to or divergent from both *C. arcticum* and *C. a.* subsp. *arcticum*. Additionally, it will be important to determine the reproductive barriers operating in both species and subspecies. Presumably, the species is self-incompatible, since most other *Chrysanthemum* species possess this reproductive barrier [39, 40]. However, if either or both of these species and subspecies were diploid it is possible they are self-compatible, which could limit gene exchange within isolated populations and lead to reduced plant height [41]. Future research on self incompatibility and ploidy is warranted.

In addition to the conservation perspective, a better understanding of variation among species and populations will facilitate the selection and use of advantageous traits. The production of chrysanthemum in the greenhouse often encounters high salinity, usually caused by the high irrigation frequency and high evapotranspiration [42]. Hence, salt tolerance in chrysanthemum becomes imperative in the response to the growing demands and the spreading application of automatic irrigation and environmental control systems [13, 24, 43, 44]. At the same time, soil salinization is a growing problem worldwide. Salt accumulation in soils is mainly derived from snow melting agents, harming the garden mums. *Chrysanthemum arcticum* and its subspecies only grow in maritime habitats throughout Alaska and Canada and acidic soils on Attu Island (Table 2), making it suited for developing salt-tolerant landscape perennials from these species and subspecies.

With the anticipated addition of *C. arcticum* subsp. *polaré* populations to the current germplasm bank, we will have a comprehensive genetic and morphological dataset. A series of studies combining the genetic (SNP marker) and phenotypic datasets would be expected. Previous studies showed that genome-wide association study (GWAS) is a practical approach that can associate individuals with phenotypes effectively and simultaneously detect allelic variations and candidate genes from a pre-established germplasm set [44–46]. Sequencing the genomes, coupled with marker-assisted selection will become a valuable tool in furthering research on the species in the *Chrysanthemum arcticum* species complex.

## Conclusions

Soil samples revealed extremely high levels of Na (≤ 2445 mg/kg) tolerated by these maritime species, which do not occur away from the oceanic coastlines; this salt tolerance is unique among *Chrysanthemum* species. Besides evolutionary phenotypic characteristics, the significant flowering differences that occurred among the species has led to further study of *C. arcticum* flowering. It is postulated that Na in soils or maritime salt spray may induce flowering in *C. arcticum,* which is a study in progress. Novel diagnostic traits of inflorescence length and plant height were discovered among *C. arcticum* and *C. a.* subsp. *arcticum* could be evolutionarily adaptable to the severe weather conditions in the Aleutian Island (Attu), where shorter inflorescence lengths may have adaptive significance [47]. This study provides insights into the extent of potential genetic bottlenecks within and among Arctic *Chrysanthemum* species. This study on the species’ variation in morphological and diagnostic traits is of importance to link morphological traits with single nucleotide polymorphism (SNP) markers. Genetic cluster analysis for *C. a.* subsp. *arcticum* verified the possibility of a higher frequency of gene flow among Attu island collection sites.

## Methods

### Study sites

This study focused on extant *C. arcticum* collected by Dr. Neil Anderson (University of Minnesota) during 2017–2018 from the coastline of southwest Alaska mainland (59° 46'N to 61° 6’N, -146° 16’W to -151° 51’W) and *C. arcticum* subsp. *arcticum* collected from the coastline of the westernmost Aleutian Island, Attu Island (52° 48’N to 52° 50’N, 173° 9’E to 173° 18’E) (*cf.* Fig. 1, [19]). There were four collection sites on the Alaska mainland for nine extant *C. arcticum* populations (n = 225 individuals in total; Table 2 *cf.* Fig.2, [19]): Anchor Point (n = 1 population), Kenai (n = 3 populations), Ninilchik (n = 1 population), and Old Valdez (n = 4 populations) (*cf.* Fig. 2, [19]) and 21 collection sites on Attu island along the coastline for 21 extant *C. a.* subsp. *arcticum* populations, Attu-1 to Attu-21 (n = 326 individuals in total; Table 2 *cf.* Fig. 3, [19]). All *C. arcticum* populations were in full flower at the time of collection (July, 2018) whereas all *C. a.* subsp. *arcticum* populations were only vegetative at the time of collection (May–June, 2018). Attu Island is the western-most Aleutian Island of North America [47, 48] and is generally classified as an Arctic [49] or Hypo Arctic zones [50]. The climate on Attu island is cool (3.8 °C mean annual temperature) with 90% of the days having measurable precipitation (average rainfall = 1,372 mm/yr.) [51]. Clones (ramets) of each ortet growing in the wild were collected for this study and were identical to those used to generate SNPs [19, 20]. Taxonomic identification occurred using dichotomous keys in the wild by Dr. Neil Anderson with flowering plants (*C. arcticum*) whilst only vegetative specimens of *C. arcticum* subsp. *arcticum* were collected in the wild. Thus, subsequent flowering and identification of *C. arcticum* subsp. *arcticum* occurred after collection with flowering specimens one month later in the greenhouse. Identifications were confirmed by curators of the Bell Museum Herbarium (MIN; University of Minnesota). One specimen of each population was deposited as a voucher specimen for future study with the following herbarium voucher specimen identifiers: MIN 2,744,013, MIN 2,744,011, MIN 2,744,010, MIN 2,744,007, MIN 2,743,973, MIN 2,743,974, MIN 2,744,006, MIN 2,744,003, MIN 2,744,000, MIN 2,743,997, MIN 2,743,994, MIN 2,743,990, MIN 2,743,987, MIN 2,743,985, MIN 2,743,983, MIN 2,743,981, MIN 2,743,979, MIN 2,743,978, MIN 2,743,977, MIN 2,743,977, and MIN 2,743,975.

### Germplasm

Where necessary, collection permits were issued for the collection and research of *C. arcticum* germplasm and soil sampling (USFWS No. 74500–17-018). In 2018, 225 individuals of *C. arcticum* were collected from the nine populations and 326 individuals of *C. a.* subsp. *arcticum* were collected from the 21 populations (Table 1 *cf.* Fig. 3, [19]). These plants were collected as rhizomes (*C. a.* subsp. *arcticum* individuals were vegetative whereas all *C. arcticum* were flowering and the complete flower stems were brought to the lab). In addition, bagged in resealable plastic bags (1.75 mil, 1 Quart Get Reddi® Reclosable Food Service Bags, https://www.usplastic.com/catalog/item.aspx?itemid=128308&catid) and put on ice in a portable cooler. Samples were placed in a refrigerator (~ 3–5 °C) until eventual transport to the lab at the University of Minnesota (within 2–3 wks. after collection, Year 1). Rhizomes were subsequently transplanted and rooted in the mist house, with an intermittent mist system (10 min of frequency; reverse osmosis water). Since the *C. arcticum* individuals were harvested with the flowers, reproductive data (with the exception of the number of days to visible bud date and flowering) was collected from them prior to rooting. The flower stems were then removed and placed into floral preservative for seed ripening (for use in subsequent experiments). After rooting for 1–2 wks., plants were moved to an environmentally controlled glass greenhouse with a 24.4 ± 3.0/18.3 ± 1.5 °C day/night daily temperature regime and a 16 h photoperiod (0600–2200 HR; long days). During the winter months, supplemental lighting was applied with 400 w high pressure sodium high intensity discharge (HPS-HID) lamps, at a minimum of 150 μmol m^−2^ s^−1^ at plant level. The computerized greenhouse was in the St. Paul campus Plant Growth Facilities (University of Minnesota, St. Paul, MN). Fertigation water was applied twice daily, between 0700–0800 HR and 1600–1700 HR, using a constant liquid feed (CLF) of 125 ppm N supplied from a water-soluble 20 N–4.4P–16.6 K fertilizer (Scotts, Marysville, OH). Monthly rotational fungicide drenches were administered (*cf* [19]*.*).

### Soil sampling

Soil samples were collected from all mainland Alaska (Anchor Point-1, Kenai-1, Kenai-2, Ninilchik-1, Old Valdez-1, -2, -3, -4) populations for *C. arcticum* individuals and one *C. arcticum* subsp. *arcticum* sample was collected from Attu island, population 10 (weight limit restrictions limited sampling all of the 21 populations, due to the need to transport via boat on the Bering Sea). Soil samples from mainland Alaska and Attu Island were collected at the base of the first plant collected, with a 250 g sample collected as topsoil subtending the existing plant material. Samples were returned to the lab in resealable plastic bags (1.75 mil, 1 Quart Get Reddi® Reclosable Food Service Bags, https://www.usplastic.com/catalog/item.aspx?itemid=128308&catid) and kept at 3-5C until submitted for Spurway Greenhouse, Florist, & Nursery Crops testing at the Department of Soil, Water and Climate’s University of Minnesota Soil Testing Laboratory (http://soiltest.cfans.umn.edu/) to determine nutrient and other factors of the native soil for species and subspecies. Soil samples were evaluated for NO_3_^−^N (mg/kg soil), SO_4_^−^S (mg/kg soil), Bray P (mg/kg soil), NH_4_OAc-K (mg/kg soil), organic matter or LOI OM (%), water pH, 1:1 electrical conductivity or EC (mmhos/cm), saturated paste extract EC (mmhos/cm), hot water boron (mg/kg soil), DTPA-Fe (mg/kg soil), DTPA Mn (mg/kg soil), DTPA Zn (mg/kg soil), DTPA Cu (mg/kg soil), exchangeable NH_4_OAc-K (mg/kg soil), NH_4_OAc-Ca (mg/kg soil), NH_4_OAc-Mg (mg/kg soil), and NH_4_OAc-Na (mg/kg soil). The NH_4_OAc-Na (mg/kg soil) determined salt concentrations rather than just EC values since ECs represent dissolved solutes, including Na.

### Measurement of phenotypic traits

**Fig. 4:**
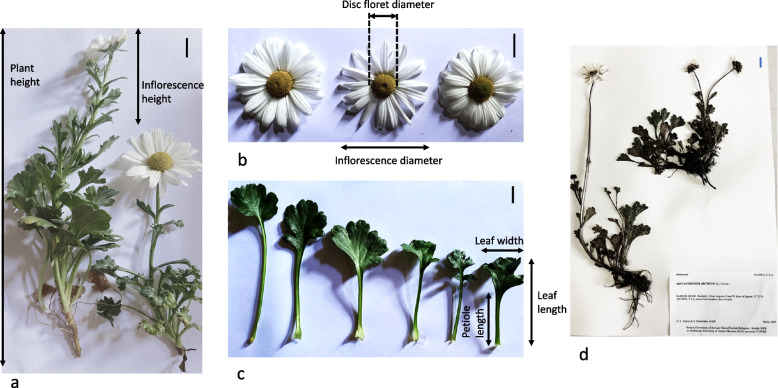
Survey standard of extant specimens for measuring above-ground plants parts of **a**) plant height, inflorescence height (*Chrysanthemum arcticum* subsp. *arcticum*, Attu-8 [left] and -3 [right] populations); **b**) inflorescence and disc floret diameters with ray petal morphological differences; **c**) petiole and leaf lengths, leaf or lamina widths; d) an herbarium specimen of *C. arcticum* from Kodiak Island, AK (University of Alaska Museum, ALA #V155200, Kodiak, AK). Photo credits: Neil Anderson. Scale: bar = 2.0 cm

The phenotypic (morphological) characteristics investigated were based on the Chrysanthemum Test Guidelines criteria set by the International Union for the Protection of New Varieties of Plants and Plant Identification Terminology [18, 21, 22]. To obtain comprehensive morphological traits datasets for *C. arcticum* populations, the same clones were grown in Year 1 (2018; from rooting onwards) through Year 2 (2019) to create data sets. In Year 1, only ramets of *C. arcticum* subsp. *arcticum* flowered (Table 3) which limited the data collection of flower data for *C. arcticum* (0% flowering)*.* Thus, the experiment was continued into Year 2 (after 6 wks. or 1000 h of cold at 3–5 °C [14]) in the event that any of the *C. arcticum* clones would subsequently flower. In the event that these did not flower in Year 2, most reproductive traits (with the exceptions of the number of days to visible bud date and flowering) were measured on the flowers collected originally on site (see above). In Year 1, plant height (cm; Fig. 4a), inflorescence length (cm), number of leaves on the primary stem, internode length (cm), inflorescence diameter (cm; Fig. 4b), disc floret diameter (cm), petal length (cm), flower fresh weight (g), flower dry weight, water in fresh flower (Δflower weight (g) = fresh flower weight—dry weight) were recorded. In 2019 (Year 2), we added leaf morphology, leaf length (cm), lamina length (cm), petiole length (cm), leaf width (cm), leaf margin, shape and color (Fig. 4c). Plant height was measured using a standard ruler (30 cm) placed vertically from the tallest point of the canopy of an inflorescence (if flowering) or from the tallest leaf (if nonflowering) to the soil line (base of the plant). Inflorescence length was measured from the bracts to the top of the plant ([52]; Fig. 4a). A comparative dried plant sample is shown from an herbarium (Fig. 4d). The color of each leaf, flower ray floret (petals) and disc floret were determined using the Royal Horticultural Society [25] chart with the visual appearance under natural sunlight in the greenhouse. Leaf morphological data was recorded by removing a representative, fully matured leaf from each individual and taking a photo of each leaf sample which were subsequently measured in Image J software [53]. Leaf length (cm) was measured from the lamina tip to the base of the leaf where the leaf stem (petiole) ended at the node on the primary stem. Petiole length was measured from the base of the petiole (at the primary stem) to the lamina base; lamina length (cm) was obtained by subtracting leaf length from the petiole length; leaf width (cm) was measured from the widest lamina lobes. The number of leaves on the primary stem of each individual were counted and mean internode length (cm) was obtained by the following equation:

#### Mean internode length (cm) = plant height / leaf number

Leaf shapes were classified into five types: flabellate, hastate, pandurate, oblong or round [21]. Leaf margins were classified into four types: cleft, crenate, entire, lobed or tripartite.

All *C. arcticum* populations were flowering in the wild during the 2018 collecting trips, but most failed to flower as clones thereafter in the greenhouse (Years 1–2; Table 2) despite being under, presumably, the correct photoperiod of long days to induce flowering. For *C. a.* subsp. *arcticum,* visible bud and flowering dates were recorded. In Year 1, the number of days to visible bud date (VBD) was counted from the day plants were rooted in the greenhouse whereas in Year 2, it was the date they were taken from the cooler after a six-wk. cold treatment (3–5 °C; [14]), to the day when terminal flower bud was visible. The number of days to flowering was counted from the same start date each year to the day the flower expanded to the widest diameter and was at anthesis (pollen shed).

Flower morphological traits were observed and recorded mainly during the mature flowering period (Year 1 data set for *C. arcticum* and Year 2 data set for *C. a.* subsp. *arcticum*). Inflorescence diameter was measured by standard ruler from the widest points of the flower. Disc floret diameter was measured from the widest point of the yellow floret disc. The petal length (cm) was calculated by the formula:

#### Petal Length (cm) = (inflorescence diameter—disc diameter)/2

The first flower on each individual was cut and weighed for fresh weight (g), placed in a high temperature oven (76.67 °C) (Hotpack, Philadelphia, PA) for 24 h, and then weighed to obtain dry weights (g). To calculate the Δ flower weight or water content, the following equation was used:

#### Δ flower weight (g) = fresh weight (g)—dry weight (g)

### Data analysis

We conducted a series of statistical analyses to evaluate the morphological characteristics and establish phenotypic relationships between species and subspecies and among populations. We used multivariate approaches to quantify the variance for each trait as well as qualify visible attributes (color) of *C. arcticum* and *C. a.* subsp. *arcticum*. We also used univariate and multivariate regression approaches based on previous studies [54, 55] to analyze the morphological characteristics among the nine extant *C. arcticum* populations and 21 extant *C. a.* subsp. *arcticum* populations, respectively. Three taxonomic groupings were established for the statistical analyses: Group one: species, subspecies; Group two: *C. arcticum;* Group three: *C. arcticum* subsp. *arcticum*. In our study, statistical analyses were conducted to detect the variation among the three taxonomic groupings: Groups one, two and three respectively, by considering corresponding quantitative morphological variables for individuals in each group. For Group one, the group label was set as *C. arcticum* and *C. a.* subsp. *arcticum*; 16 quantitative morphological variables were applied using PCoA. For Group two, the group label was set at nine extant populations of *C. arcticum*; 16 quantitative morphological variables were analyzed with PCoA. For Group three, PCoA was used to detect the differences among the 21 populations of *C. a.* subsp. *arcticum* by analyzing 16 quantitative morphological variables. Collected quantitative morphological data were analyzed and performed using R studio (v.1.3.959) and the Statistical Package for the Social Sciences (SPSS) software, v.25.0 [56].

Two multivariate analyses, principal component analysis (PCoA) and Pearson’s correlation were performed. The PCoA is one of the most effective and frequently used multivariate statistical methods for investigating a large set containing individuals/entities of multiple inter-correlated variables [57, 58]. PCoA reduces the dimensionality of a multivariable dataset to few new variables, termed principal components, which correspond to a linear combination of the original variable [58]. Each principal component was reassigned a different portion of original variables, whereby PC1 would be considered as the greatest weight, PC2 would be the second, etc. [55]. Principal components analyses for three groups (Group one, two, three) were conducted with R studio by using the FactoMineR [57] and factoextra R packages [59]. The relatedness between morphological traits among populations for each group were assessed using Pearson’s correlation coefficients and tested at *p* ≤ 0.05 and *p* ≤ 0.01 [60].

Univariate Analysis of Variance (ANOVA; general linear model) and descriptive statistics were conducted using SPSS to identify the discriminative descriptors and statistically differentiate among populations for quantitative phenotypic characteristics. Mean separations were conducted using 5% Tukey’s Honestly Significant Difference (HSD) test at α = 0.05. The ANOVA analyses applied to *C. arcticum* and *C. a.* subsp. *arcticum* species, separately. While the *C. arcticum* dataset was combined by using both data from year 2018 and 2019, *C. a.* subsp. *arcticum* only included the data from 2019. The morphological variables from different years and different species would influence the univariate in the analyses. Hence, the comparison between species and subspecies would not be included in the univariate analysis of variance. The variation within and among populations of *C. arcticum* and *C. a.* subsp. *arcticum* between leaf quality morphological variables (leaf shape and leaf margin) was compared by a Chi-square (χ2) test for equal distribution across the five classes for the leaf shape and leaf margin data (1:1:1:1:1χ2).

## Data Availability

The datasets used and/or analyzed in the current study are available from the corresponding author on reasonable request.

## Abbreviations

ANOVA: Analysis of variance
Ca: Calcium
Cu: Copper
DAPC: Discriminant analysis of principal components
DArTseqLD: Diversity arrays technology low density sequencing
EC: Electrical conductivity
Fe: Iron
GWAS: Genome wide association mapping
H_o_: Null hypothesis
HSD: Tukey’s honestly significant difference test
K: Potassium
Mg: Magnesium
mmhos/cm: Millimhos per centimeter
Mn: Manganese
N: Nitrogen
Na: Sodium
NH_4_OAc-Na: Ammonium acetate
NO_3_-N: Nitrate nitrogen
OAc-Na: Sodium acetate
P: Phosphorous
PC: Principal component
PCoA: Principal components analysis
SNP: Single nucleotide polymorphism
SPSS: Statistical package for the social sciences
SS: Soluble salts
UPGMA: Unweighted pair group method with arithmetic mean
VBD: Visible bud date
χ2: Chi-square
Zn: Zinc
